# Neural networks for open and closed Literature-based Discovery

**DOI:** 10.1371/journal.pone.0232891

**Published:** 2020-05-15

**Authors:** Gamal Crichton, Simon Baker, Yufan Guo, Anna Korhonen

**Affiliations:** Language Technology Laboratory, TAL, University of Cambridge, Cambridge, United Kingdom; Universidad de Jaen, SPAIN

## Abstract

Literature-based Discovery (LBD) aims to discover new knowledge automatically from large collections of literature. Scientific literature is growing at an exponential rate, making it difficult for researchers to stay current in their discipline and easy to miss knowledge necessary to advance their research. LBD can facilitate hypothesis testing and generation and thus accelerate scientific progress. Neural networks have demonstrated improved performance on LBD-related tasks but are yet to be applied to it. We propose four graph-based, neural network methods to perform open and closed LBD. We compared our methods with those used by the state-of-the-art LION LBD system on the same evaluations to replicate recently published findings in cancer biology. We also applied them to a time-sliced dataset of human-curated peer-reviewed biological interactions. These evaluations and the metrics they employ represent performance on real-world knowledge advances and are thus robust indicators of approach efficacy. In the first experiments, our best methods performed 2-4 times better than the baselines in closed discovery and 2-3 times better in open discovery. In the second, our best methods performed almost 2 times better than the baselines in open discovery. These results are strong indications that neural LBD is potentially a very effective approach for generating new scientific discoveries from existing literature. The code for our models and other information can be found at: https://github.com/cambridgeltl/nn_for_LBD.

## Introduction

Literature-based Discovery (LBD) aims to discover new knowledge by connecting information which have been explicitly stated in literature to deduce connections which have not been explicitly stated. Its pioneer is Don Swanson who hypothesised that the combination of two separately published results indicating an A-B relationship and a B-C relationship are evidence of an A-C relationship which is unknown or unexplored. He used this to propose fish oil as a treatment for Raynaud syndrome due to their shared relationship with blood viscosity [1]. This hypothesis was later shown to have merit in a prospective study [2] and he continually proposed other discoveries using similar methods [3–5]. LBD comes in two flavours: open and closed discovery. In open discovery, only the A is given and Cs are deduced using the various A-B-C relationships in existence while in closed discovery the A and C are given and the goal is to quantify the existence of relevant Bs.

LBD has evolved to involve using computers to discover many such connections automatically from large collections of literature. Thus, it can facilitate both hypothesis testing and generation to give tangible support to scientific research [6, 7]. Scientific literature is growing at an exponential rate [8], making it difficult for researchers to stay current in their discipline. This, along with the increasing necessity of researchers to specialize has led to an environment where discoveries in one area are not known outside of it [9] and valuable logical connections between disparate bodies of knowledge remain unnoticed [10]. This means there is a very real chance that knowledge which can be combined to form or crystallise breakthrough-inducing hypotheses are dispersed throughout the literature. LBD can help researchers to quickly discover and explore hypotheses as well as gain information on relevant advances inside and outside of their niches and increase interdisciplinary information sharing. Thus as the scientific literature grows, the necessity for LBD as a research tool increases.

LBD has already been used to identify new connections between biomedical entities and new candidate genes and treatments for illnesses [6] and to propose treatments for Parkinson’s Disease and Multiple Sclerosis [11, 12]. It has seen use in drug development and repurposing [13, 14] as well as predicting adverse drug reactions [15, 16]. It has also been used to propose new potential cancer treatments [17] and identify promising research collaborations [18].

The recently-released LION LBD system [19] reports state-of-the-art results in LBD. It uses PubTator [20] for annotating PubMed scientific articles with concepts such as chemicals, genes/proteins, mutations, diseases and species; as well as sentence-level annotation of cancer hallmarks that describe fundamental cancer processes and behaviour [21]. It uses co-occurrence metrics to rank relations between concepts and performs both open and closed discovery.

Neural networks have been successful in related tasks such as Knowledge Discovery and Natural Language Process (NLP) in recent years. Whether they can be used to give improved results in LBD is unexplored (except for recent exploratory work by [22]). In this paper we make two main contributions: four graph-based neural approaches to LBD; and evaluations of them on two real-world biomedical datasets using informative metrics. These datasets tested their ability to rank future published biomedical discoveries: one is the Cancer Discovery dataset used by [19] and the other consisting of human-verified, peer-reviewed biomedical interactions.

## Related work

### Literature-based Discovery (LBD)

LBD seeks to discover previously unknown associations or hidden links between pieces of existing knowledge by analysing literature in an automated or semi-automated way using various computational approaches and algorithms [23, 24]. It has mostly been deployed in the biomedical domain, but it has also been used outside of it as it has been applied to research into developing water purification systems, accelerating development of developing countries and identifying promising research collaborations [18, 25, 26].

[1] defined the most basic and widespread type of LBD, called the ABC paradigm because it centres around three concepts called A, B and C (e.g. [27–29]). It states that if there is a connection between A and B and one between B and C, then there is one between A and C which, if not explicitly stated, is yet to be explored. The ABC paradigm has two types: *open* and *closed* discovery.

In open discovery, only A is given. The approach finds Bs and uses them to return possibly interesting Cs to the user, thus *generating hypotheses* from A. With closed discovery, the A and C are given to the approach which seeks to find the Bs which can link the two, thus *testing a hypothesis* about A and C.

[30] distinguishes between traditional approaches and ‘emergent’ paradigms which will define the field in the future (e.g. [31, 32]). One of the characteristics of these is their use of techniques borrowed from other research fields including link prediction on graphs and machine learning which offer different approaches to LBD and address its problems. This work provides a blend by using the ABC paradigm but harnessing machine learning models inspired by link prediction on graphs.

### Evaluating LBD systems

Evaluation is difficult in LBD for several reasons: disagreement about the role of LBD systems in research and thus what makes a successful one; difficulty in determining how useful, interesting or actionable a discovery is; and difficulty in objectively defining a ‘discovery’, which hinders the creation of a standard evaluation set which quantifies when a discovery has been replicated or found. Nonetheless, several methods have been employed in previous work.

A popular methods used in LBD is to *replicate previous discoveries* [28, 33, 34]. These are usually LBD-based discoveries as they are relatively easy to quantify compared to other discoveries. This means that there are only a handful of such discoveries and there is a danger of designing approaches which are tuned to perform well on these discoveries but do not generalise. In this evaluation, the literature before the discovery to be replicated is used to generate a ranked list of discovery candidates as target or linking terms. Success is measured by reporting the rank of the term(s) of interest; the higher the rank, the better the approach.

*Literature- or time-slicing* involves splitting the existing literature at a point in time. The approach is then exposed to the literature before the split and is evaluated by how many of the discoveries in the later period it can discover. Unclear definition of a discovery and an inability to determine if a discovery is wrong or simply new are critiques of this approach. In the absence of a perfect gold standard, this approach estimates it by finding instances of the defined relationships in the test set which are not in the training set and can be reasonably inferred from it. This means that evaluation depends on what constitutes a relationship for the given dataset. If a noisy relationship is used, the evaluation will be easy to perform well on. Previous systems have used term co-occurrences [35], relationships from external biomedical resources (e.g SemMedDB) [32] and semantic relationships [36]. A high precision approach would be to get expert opinion to generate the gold standard [37], but this is time-consuming, expensive and tends to produce low recall rates.

The advantage of this evaluation is that it produces an indicator of an approach’s performance on a large number of test instances. This raises the need for evaluation metrics which can quantify performance on large, ranked lists. LBD works have used metrics popular in Information Retrieval [38] which include Precision, Recall, Area Under the Curve (AUC), Precision at *k*, Mean Average Precision (MAP) etc.

*Proposing new discoveries* or treatments goes beyond replicating past discoveries or predicting time-sliced instances of a particular relationship and shows that a system is capable of being used in realistic situations [13, 33, 39, 40]. This is usually accompanied by peer-reviewed publication in the domain or vetting by a domain expert.

### Neural networks in the biomedical domain

While they are yet to be applied to LBD, the versatility of neural networks have been shown in their application to a broad range of biomedical tasks. They have been used to predict mental health conditions from tweets [41], recognise biomedical entities in text [42, 43], classify hallmarks of cancer in text [44] and predict links representing Drug-Target Interactions (DTIs) and Protein-Protein Interactions (PPIs) in biomedical graphs [45]. On the biomedical image front, they have been used for classifying biomedical images [46, 47], segmenting 3D biomedical images [48] and segmenting and enhancing cardiac images [49].

An excellent recent overview of the use of neural networks in the biomedical domain is [50]. They point out that beyond the well-known applications to diagnosis, neural networks are increasingly being used to inform healthcare management decisions.

### Node representations as embeddings

Graphs encode knowledge and can be processed to extract information which may not be easily seen. For machines to process them, graphs must be represented in a useable format, usually representing nodes as vectors of real numbers. Research on node representation devises methods which can create representations which preserve the original information in the graph. This information relates to the nature of the links and are classified as first or second (or higher) order proximity [51, 52]. Given two nodes, first order proximity is concerned with the strength of the direct link between them. Second order proximity compares their neighbourhoods and classes them as similar if their neighbourhoods are similar.

The quality of a method depends on its ability to preserve the proximities of a graph when creating representations. The node representations created by recent research represents each node as a vector in a space where similar nodes are located close to each other (node *embeddings*). There has been a proliferation of methods to create these node embeddings from graphs and it would be unwieldy to include all of them in this work. Comparisons between some of these can be found in [52]. We utilised a popular method whose implementation is freely available online, supported weighted edges and scaled to our large graphs: Large-scale Information Network Embedding (LINE) [51].

LINE explicitly defines two optimization functions to capture the structure of the graph. One captures first order proximity and the other captures second order proximity. [51] report that training their model with each setting then concatenating the outputs gives the best performance.

## Materials and methods

### Evaluation

Here we discuss the method of preparation of the datasets used for LBD and the metrics used for evaluation. The datasets contain information on the year that each link in the graphs was formed and the graphs were split by year of link formation for training and evaluation. The methods were given the earlier links and asked to predict later links.

#### Cancer case discoveries

To facilitate direct comparison, we evaluate on the cases used in [19], which describes them at length. For completeness, we provide a summary. They are a set of five triples that represent specific recently-published discoveries (2011-2016) on the molecular biology of cancer that could have potentially been suggested by an LBD system in the past. They were selected and curated by cancer biologists. There are an additional five pairs of discoveries proposed by Swanson. The B connection was not simple in the Swanson cases so it was not possible to create triples to facilitate performing closed discovery on those cases. The details of these can be found in the Supplementary Document (S1 File) which accompanies this paper.

Each LBD approach is given a graph constructed only from literature up to five years before the publication date of the discovery and the model is then given the A and C nodes (in closed discovery) and asked to rank the B nodes. In open discovery it is given only the A node and asked to rank all nodes within two hops (the C nodes). The approach’s performance is quantified using the rank of the gold response in the returned list.

#### Time-slicing

The Cancer Discovery cases described above are strong evaluations for biomedical LBD systems because showing how a system would have ranked a discovery later published in a top-tier, peer-reviewed journal is a potent argument for its usefulness for LBD. However, the dataset is unsuitable for machine learning because it does not provide a development set to tune hyperparameters on; neither is it obvious how to create one. This, in addition to its limited size prompt the need for additional evaluation methods to gain a more generalised picture of performance of our approaches and models.

For this we choose a dataset which contained human-curated biomedical interactions which were published in peer-reviewed journals (details in “Datasets” Section). A graph created from the interactions in this dataset is time-sliced. From the post cut-off publication year, development and test sets are constructed. In some senses, this is not as stringent an evaluation and it is not possible to do closed discovery with it, but this provides robust additional evaluation of our open discovery approaches on a larger test set which is more indicative of approach generalizability.

#### Metrics

The evaluation metrics are important when analysing the performance of ranking systems. [19] reported median ranks over the groups of cases for the case discoveries. For comparability, we also report this along with the mean over the cancer and Swanson cases separately and combined.

For the time-sliced experiments, we additionally report MAP, Mean Reciprocal Rank (MRR) and Mean R-precision. There are 2 reasons for this: there is great variance between the amount of Cs which are ranked for each A so the mean rank can vary widely, distorting the results; and these metrics, especially the latter 2, give higher priority to correct scores ranked highly in the list, which is of importance in any ranking problem but especially so for LBD where investigating each proposal is a costly endeavour. Formal definitions of these evaluations are in Section 2 of the Supplementary Document (S1 File) which accompanies this paper.

### Baselines

The baseline approaches are those used by [19]. They are 8 co-occurrence metrics accompanied by three aggregator functions and two accumulator functions (explained later in this section). We present a condensed version here for completeness (names in brackets are the shorthand they will be referred to going forward). More details can be found in the referred paper: Section 3.3 and full details in its Supplementary Information document.

- Co-occurrence count (Count): the number of sentences in which mentions of the entities connected by the edge co-occur.
- Document count (Doc-count): the number of documents in which mentions of the entities connected by the edge co-occur.
- Jaccard Index (Jaccard): the ratio of the size of the intersection over the size of the union of the sets of sentences in which the entities occur.
- Symmetric conditional probability (SCP): the product of the conditional probabilities of one entity being mentioned in a sentence where another occurs.
- Normalized pointwise mutual information (NPMI): a measure of the independence of the mention occurrence distributions,
- Chi-squared (*χ*^2^), Student’s *t*-test (*t*-test) and log-likelihood ratio (LLR) are statistical tests measuring whether the mention distributions are independent of each other.

A number of alternatives for the scoring functions operating over the edge weights have also been implemented. For the aggregation function *f(g)*, the alternatives *min*, *avg*, and *max* are used. These functions assign the score for a path the minimum, mean, and maximum respectively of the edge weights on the path. For the accumulation function *f(c)*, the choices *sum* and *max* are supported. When multiple paths lead to the same node, the former sums the path score to obtain the node score while the latter simply uses the maximum score.

We focus on only the best performing methods for the mean and median metrics and report the relevant accumulator and aggregator functions in each experiment.

### Neural approaches

Two neural link prediction methods are used for closed discovery and another two for open discovery. All approaches use node embeddings created with LINE with weighted edges, where weights are calculated using Jaccard Index. The embeddings were induced with the portion of the graph used for training, the pre-cutoff year period. The settings used are in Section 3 of the Supplementary Document (S1 File).

For each of the approaches described here, five node combination methods are used to determine how the nodes which constitute the link path are combined for input into the model, so models ending in ‘-A’ refer to approaches which use Average to do this, ‘-C’—Concatenation, ‘-H’—Hadamard, ‘-W1’- Weighted-L1 and ‘-W2’- Weighted-L2.

#### Closed discovery neural model and approaches

In both of these approaches the model is a Multi-Layer Perceptron (MLP) which was effective in the similar task of neural link prediction on biomedical graphs [45]. The model contains a single hidden layer with ReLU [53] activation which led to a final layer with Softplus activation to allow for unrestricted positive scores. The model is trained as a classifier with the Cross Entropy loss.

As we use the model from [45], it is necessary to distinguish that work from this one. That paper presents a neural architecture for classifying whether a link exists between 2 nodes using their node embeddings; such an approach is not ABC LBD as is the focus of this paper. To perform ABC LBD the path(s) between A and C must be taken into account, which link prediction as proposed in [45] is unconcerned with. In this paper, the paths are taken into account in 2 different ways as reflected in the 2 approaches to Closed Discovery whose descriptions follow.

**CD-1**: The neural model is used to provide a score for each A-B or B-C link in the path. The scores are then used in aggregator functions as the baseline methods, so the difference here is that a neural network produces a score for the link instead of using one of the metric calculations described in ‘Baselines’.

**CD-2**: In this approach, A-B-C embeddings are combined to create a single input to the model which then predicts a score for the entire A-B-C link. This negates the need for an aggregator function as in the baselines and CD-1 approach. This has the additional benefit of making it trivially easy to calculate a score regardless of the length of the path between A and C, by simply combining any additional node embeddings as the initial 3 and passing it to the neural network.

#### Open discovery neural models and approaches

The approaches used for open discovery are presented and explained here.

**OD-1**: The same model and a similar approach to CD-1 is used here: the neural model is used to provide a score for each A-B or B-C link in the path from A to each possible C. The scores are then used in aggregator functions. The difference in open discovery is that here the scores are then also used in the accumulator functions to rank different paths which lead to the same C.

**OD-2**: A Convolutional Neural Network (CNN) model is used to implement an approach to open discovery which removes the need for aggregator and accumulator functions. As in CD-2, all the node embeddings of a path are combined into a single vector, however as this is open discovery, there will be many paths that lead to the same C. To obtain a score which uses information from all these paths, the combined vectors are stacked to create a window which we pass into a CNN which outputs a score indicative of the strength of the A-C links. This is analogous to passing an image to a CNN, but here the ‘image’ is produced by stacking vector representations of ABC links. The convolutional filter always slides down the stack of links, never across so that it always covers the entire link. The ABC links to be stacked are combined using the same 5 link combination methods as mentioned above.

The reader will perhaps note that the CNN expects a fixed size input and the amount of paths leading to a C will inevitably vary from case to case, creating varying input sizes which could exceed a fixed window size. To deal with this, we combine multiple windows into a single window using elementwise summation. As the total number of links will not always be a multiple of the window size, zero padding is used to fill any remaining gaps. For example: if a particular case has 175 paths and an input size of 50 is used, we will be able to sum 3 windows of 50 and as there will be only 25 paths in the final input 25 more paths will be zero padded to the input to make it 50.

**Fig 1:**
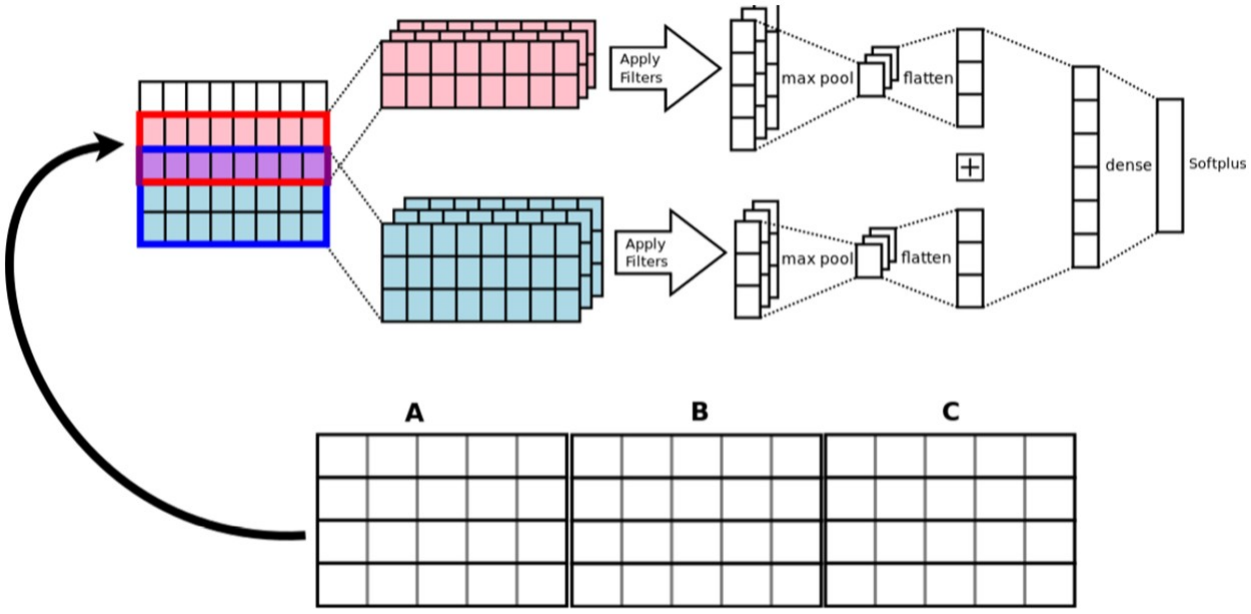
The open discovery 2 model.

In this model, the input layer leads to a batchnormed convolutional layer with ReLU activation units, then a max pooling layer then a fully connected layer before the final layer with Softplus activation. Unlike the other models which are trained as classifiers, this model uses a pointwise approach, employing Mean Squared Error (MSE) loss, to learning the ranking function by using the Jaccard Index score of the AC link as the multi-level ratings (see [54] for a more detailed description of this). The model is depicted in Fig 1.

### Datasets

The graphs we use were created from the following datasets. The graph details can be found in Table 1.

**Table 1:** Graph details (undirected link count).

Dataset	Node Count	Link Count	Link Type
BioGRID	68,734	1,209,578	Published Interactions
PubTator	∼194,691	∼12,797,468	Literature Co-occurrences

#### PubTator

Biomedical entities recognised by PubTator mentioned in the titles and abstracts of PubMed publications from 1873 to 2017 were used to create this dataset. A link exists between two biomedical entities if they co-occur in a single sentence. The annotations were downloaded on June 20th, 2017. Instances of ‘hallmarks of cancer’ which identify fundamental cancer processes, identified in text using [44] were also added to the graph as entities.

#### Biological general repository for interaction datasets (BioGRID)

This is an open database created from manually curating experimentally-validated genetic and protein interactions that are reported in peer-reviewed publications [55]. The latest release [56] includes over 1 million Genetic and Protein interactions across all major organism species and humans. Links in this graph represent biomedical interactions from published, experimentally-validated genetic and protein interactions. We use version 3.4.167 of this dataset.

## Experiments

As all approaches create ranked lists, the possibility of tied ranks exists. We use the median of the tied range to determine the rank of a gold item with ties, for example a gold ranked 10th with 10 ties is ranked the median of 10-20 range: 15th.

### Details of neural approaches

Unlike the baseline models, the neural approaches need negative examples for training. We create these by selecting either A-B or B-C links which did not form for a given A-C or A-C connections which do not exist for models which operate on the entire link path (i.e. CD-2 and OD-2).

All models are trained with batch size 100, training set size 200,000 for 150 epochs with the Adam optimiser [57], but the model is evaluated on the case after every 5 epochs and the best performance reported. For the BioGRID experiments, because evaluation is a lot more time-consuming, the models are evaluated every 25 epochs on the development set and the best performing model on MRR is evaluated on the held out test at the end. The CNN uses a learning rate of 10^−5^ while the MLPs use 10^−4^. For CD-1, CD-2 and OD-1, there is a single hidden layer with 100 units. For OD-2, the input height is 50 and the width is the size of the combined vector dimensions. The convolution window height is 7 and the convolutional output size is 128.

### Case discoveries

We use the data from [19] directly, so that our results will be directly comparable. The graphs are cut off at the relevant years before the publication date of the discovery.

#### Closed discovery on cancer discoveries

For CD-1, the model is given the A-B and B-C links and the scores it produces are used in the aggregator functions to rank the Bs. For CD-2 the model is fed all the A-B-C links for the given A and C in each triplet and the score it produces is used to rank the Bs.

#### Open discovery for cancer and Swanson discoveries

For OD-1, the model is given the A-B and B-C links and the scores it produces for each link were used in the aggregator functions to produce a score for each path. The different paths which lead to the same C are used in the accumulator functions to produce a score which is used to rank the Cs. For OD-2, the model is given all the A-B-C links for the given A and C in each pair and the score it output is used to rank the Cs.

### BioGRID

The graph is split at the year 2016. We randomly split the post-2016 links into development and test sections. The development set is used to determine which epoch has the best trained model for evaluation. Due to computational constraints, we have to restrict the amount of nodes we could evaluate on. We randomly select 1,000 entities from the test set to be A nodes and have the model score each node within two hops as the Cs. The scores are then used to rank the Cs. Like the Swanson cases, it is not possible to perform closed discovery on this dataset.

## Results

The results of the neural approaches are the median ranks and mean ranks averaged over five runs. The standard deviations reported are of the mean ranks. The results of the baselines are simply the means of the method across all relevant cases (they were not run multiple times as the neural approaches were as they are not subject to per-run variances as the neural network methods are) and the standard deviations are over those ranks.

The best score for a metric is in **bold** and the best for an approach is underlined. We sought to determine what methods gave the lowest mean ranks and lowest variance, measured by standard deviation. ‘Metric’ refers to mean and median ranks. ‘Approach’ refers to the three approaches: Baselines (Jaccard, t-test etc.), neural discovery approach 1 (CD-1, OD-1) and neural discovery approach 2 (CD-2, OD-2). Thus each ‘metric’ column should have a bolded term and each approach category (delineated by horizontal lines in the tables) should have an underlined term.

To increase clarity in the tables, we selected only the best results for each approach to show here. Full experimental results can be found in Section 4 of the Supplementary Document (S1 File). Where applicable, the accumulator and aggregator functions (explained in the “Baselines” section) are listed in the “Details” column as ‘Acc’ and ‘Agg’ respectively.

### Closed discovery on cancer discovery cases

The results for closed discovery performed on the five Cancer Discovery cases used to evaluate LION are in Table 2.

**Table 2:** Closed discovery: Mean and median ranks on the cancer discovery cases.

Approach	Mean Rank	Std. Dev.	Median	Details
**Jaccard**	214.8	256.9	81.0	Agg: min
*t* **-test**	262.0	341.8	56.0	Agg: min
**CD-1-A**	86.3	52.0	93.8	Agg: min
**CD-1-C**	94.5	80.0	**36.4**	Agg: min
**CD-2-C**	**48.7**	19.5	42.0	-

### Open discovery on cancer discovery and Swanson cases

#### Open discovery on cancer discovery cases

The results for open discovery performed on the five Cancer Discovery cases used to evaluate LION are in Table 3.

**Table 3:** Open discovery: Mean and median ranks on the cancer discovery cases.

Approach	Mean Rank	Std. Dev.	Median	Details
**NPMI**	60.2	54.4	36.0	Acc: sum, Agg: max
**Count**	367.4	553.3	15.0	Acc: sum, Agg: min
**OD-1-C**	93.4	145.8	31.4	Acc: sum, Agg: min
**OD-1-A**	218.3	368.7	26.8	Acc: sum, Agg: min
**OD-2-H**	**31.1**	11.9	**12.2**	-

#### Open discovery on Swanson cases

The results for open discovery performed on the five Swanson cases used to evaluate LION are in Table 4.

**Table 4:** Open discovery: Mean and median ranks on the Swanson cases.

Approach	Mean Rank	Std. Dev.	Median	Details
**Doc-Count**	2,199.8	4,216.7	31.0	Acc: max, Agg: avg
*t* **-test**	3,956.4	7,899.3	5.0	Acc: max, Agg: avg
**OD-1-H**	3,558.3	7,930.7	19.2	Acc: sum, Agg: min
**OD-1-C**	3,721.4	8,306.7	**4.0**	Acc: sum, Agg: min
**OD-2-H**	**1,013.4**	167.9	17.6	-

#### Open discovery on cancer discovery and Swanson cases

The results for open discovery performed across the five Cancer Discoveries and five Swanson cases combined are in Table 5.

**Table 5:** Open discovery: Mean and median ranks on all open discovery Cases.

Approach	Mean Rank	Std. Dev.	Median	Details
**Jaccard**	1,634.4	4,733.9	21.0	Acc: sum, Agg: min
**Count**	1,925.8	5,171.3	**11.5**	Acc: sum, Agg: min
**OD-1-C**	1,907.4	5,859.4	18.2	Acc: sum, Agg: min
**OD-2-H**	**522.2**	89.9	14.9	-

### Open discovery on BioGRID published interactions

Results for open discovery performed on the BioGRID dataset. Performance across the 4 metrics explained in the “Metrics” Section are in Table 6.

**Table 6:** Open discovery on time-sliced BioGRID.

Approach	MR	MRR	R- Prec.	MAP	Details
**Jaccard**	1,197.3	2.19	2.47	2.86	Acc: sum, Agg: min
**LLR**	1,132.9	1.34	1.38	1.9	Acc: sum, Agg: max
**OD-1-H**	1,907.5	0.92	0.96	1.25	Acc: sum, Agg: max
**OD-1-C**	1,913.4	0.94	1.01	1.23	Acc: sum, Agg: max
**OD-1-W2**	1,908.3	0.92	0.98	1.26	Acc: sum, Agg: max
**OD-2-C**	**1,113.1**	**3.42**	**4.73**	**5.46**	-

## Discussion

### Closed discovery on cancer discovery cases

The results of this experiment can be found in Table 2. The neural approaches performed much better than the existing methods in these experiments. Rows 3 and 4 show that the performance doubled, by halving the mean ranks, simply by replacing the baseline scoring metrics with a small neural classifier to provide the scores instead. It almost doubled again by replacing the aggregation of individual path scores with combining the vectors of the nodes involved in the path (row 5). Performance on the median also increased though not as drastically.

Of note here is that the neural approach which dispelled with the aggregator functions, instead opting to combine the inputs and obtaining a score for the entire path, was the best performer on mean ranks and the second best performer on median (row 5). This indicates that the information which the aggregator functions seek to provide to an approach is better provided by combining the vector representations of the nodes in the path.

### Open discovery on cancer discovery and Swanson cases

#### Open discovery on cancer discovery cases

The results of this experiment can be found in Table 3. Despite the strong improvements seen in closed discovery by simply replacing the baseline scoring metrics with a neural classifier, that was not the case here for either mean or median rank (rows 3-4). However, the more complex CNN approach was able to produce results which approximately doubled performance on mean rank from a strong baseline (row 5). It also performed the best on median rank.

Analogous to the closed discovery experiments, the approach which dispelled with aggregators and accumulators outperformed on mean ranks (row 5). Additionally, it was the best median performer here, further validating it.

#### Open discovery on Swanson cases

The results of this experiment can be found in Table 4. A similar trend to the cancer cases was shown here: simply replacing the baseline scoring metrics with a neural classifier decreased performance on mean rank, although one such approach did perform the best on median rank (rows 3-4). The strong performance of the CNN continued as it again doubled performance on mean rank although it was only the third best on median rank (row 5). The trend of the approach which dispelled with aggregators and accumulators outperforming on mean ranks also continued.

#### Open discovery on both cancer discovery and Swanson cases

The results of this experiment can be found in Table 5. Given the results of the subset experiments, it is not surprising that the CNN was the best performer across all open discovery cases (row 4). Its performance on mean rank was approximately three times better than that of the best baseline and it was the second best on median, although the simple count baseline approach was the best.

#### General open discovery

In addition to its strong performance across the cases (Tables 3, 4 and 5), the OD-2-H approach is also the most stable as it showed the lowest variation in performance over multiple runs of the best performing methods as measured by the standard deviation shown in those tables.

A point in favour of the neural approaches over the baselines is their apparent consistency in performance over the subsets of the cancer and Swanson cases. The baseline methods which performed the best, shown in Tables 3, 4 and 5, varied while the best neural approaches recurred, demonstrating their invariability to the vagaries of the case subsets.

### General case discoveries

Whether to use mean or median as average for these experiments is a valid question. [19] reported median and we do the same to allow for comparison, but also report the mean because we believe that it is better suited to this situation. The median is robust to outliers and can give a more accurate picture of a system’s performance when an outlier can radically affect the mean, as is the case with the Swanson cases used. However, the aim of this research is to find an approach which will aid researchers on totally novel data, so the worst-case performance of the system (even if it is rare) is of importance and the aim should be to use methods which give the best results across all cases. Thus, evaluating accurately should involve looking at performance in all available cases. Median ignores not only outliers, but effectively all performances beyond the median (approximately half the use cases). The argument can thus be made that the median does not give an accurate reflection of an approach’s performance.

Taking mean as a preferable metric to median, the case of the neural methods is strengthened as they were the best performers across all the case experiments. Additionally, there was low variance among the best neural approaches. It was also pleasing to find that approaches which dispelled with the cumbersome aggregator and accumulator functions were the best. This indicates that when given the full path information, the neural models are able to discern how best to use it to improve performance.

It is also noteworthy that although methods which concatenated the node representations performed well, there were other approaches whose performance were comparable or better than it across these experiments. This is significant because unlike the concatenate combination method, which increases the input size linearly with the path length, the other node combination methods keep a fixed input size which makes them indifferent to the amount of hops between A and C. This feature makes them amenable to approaches to LBD beyond the simple two-hop ABC paradigm to the *n*-hop *AB*_1_ *B*_2_…*B*_*n*_ *C* paradigm which it is generally agreed must be overcome for LBD to reach its true potential.

### Time-sliced BioGRID

The reasons for undertaking these experiments were explained in the “Time Slicing” Section and the reasons for the multi-faceted evaluation in the “Metrics” Section. We will make use of and expand on these here.

The data used in this experiment represent experimentally-validated, human-curated interactions which were published in peer-reviewed publications. Thus the knowledge proposed by using it is of high quality. Additionally, the evaluation is time-sliced which is reflective of how knowledge discovery progresses in the real world and involves far more evaluation instances than a handful of cases, notwithstanding the very high quality of the cases.

LBD across a large amount of possible positives is a ranking problem because its proposals are usually costly to investigate. Thus priority should be given to approaches which can rank correct new associations at the very top of the list even if they rank more of them lower; the classic precision-recall trade-off. Performance too far down the list can effectively be ignored: when experimentally validating new knowledge proposals, whether it is ranked 200^th^ or 900^th^ is likely of little concern to a user; it is too far down the list.

Metrics like MAP, MRR and R-precision place value on higher ranked true positives but they do not do so equally. MAP and MRR are concerned with the entire list but MRR punishes lower-ranked correct items more when the retrieval space is large as it tends to be in LBD, especially open discovery. R-precision literally discards most of the returned results and reports results only on the best. Thus performance on metrics like R-precision and MRR give a better idea of the practical worth of an LBD system, especially on open discovery.

The results of this experiment can be found in Table 6. The OD-2-C method we introduce here performs approximately 1.5-1.9 times as good the baseline approaches on these metrics, in addition to strong performance on MAP and mean rank (row 6). It is a variant of the OD-2-H method which showed vastly better performance on the cases experiments. The results here thus validates the OD-2 (CNN) approach to open discovery we presented in the Section “Open Discovery neural models and approaches”.

The role of the node embeddings in the superior performance of the neural network methods may be difficult to isolate but we can surmise how they can contribute. The node embeddings utilise both first order and higher order proximities which incorporate information from a node’s wider neighbourhood than the baseline scoring methods would. This additional information can aid in ranking a node and lead to improved performance.

While there is still lots of room for improvement, these results are dependable and demonstrate the potential for using neural networks to perform even traditional open and closed discovery within the ABC paradigm.

## Conclusion

LBD aims to discover new knowledge automatically from large collections of literature. Scientific literature is growing exponentially, making it difficult for researchers to stay current in their discipline. LBD can solve this problem by facilitating hypothesis testing and generation to give tangible support to scientific research.

We proposed four neural network-based approaches to open and closed LBD. We compared our methods with those used by a state-of-the-art LBD system to replicate recently published findings in cancer biology and also applied them to a time-sliced dataset of human-curated, peer-reviewed biological interactions. In both cases, our methods showed a notable and significant improvement over the existing methods on metrics adapted to the situation.

Although there is scope for much improvement, these results strongly demonstrate the potential of using neural networks to perform open and closed LBD well within the ABC paradigm and in some cases using only sentence-level co-occurrence relationships. Combined with previous work on the viability of using neural link prediction for LBD, they indicate that neural networks can significantly improve performance on the increasingly important task of LBD. Immediate future work includes using the pairwise approach to learning the ranking function for the CNN approach, using more advanced graph embedding techniques to better capture the information present in graphs and applying attention to the neural approaches to determine which paths are contributing the most to its performance.

## Supporting information

S1 File Supplementary document. Contains additional results and formal definition of evaluation metrics which were left out of the paper in pursuit of conciseness.
(PDF)
