# Supplementary material for: Neural networks for open and closed Literature-based Discovery
Source: PLoS One. 2020 May 15;15(5):e0232891. doi: 10.1371/journal.pone.0232891 (PMC7228051; doi:10.1371/journal.pone.0232891)
Supplement: S1 File — Contains additional results and formal definition of evaluation metrics which were left out of the paper in pursuit of conciseness. (PDF) [file pone.0232891.s001.pdf]

Supplementary Document for 'Neural networks for open and closed Literature-based discovery'

Gamal Crichton, Simon Baker, Yufan Guo and Anna Korhonen

1 Introduction

This document is a supplementary to the paper: *Neural networks for open and closed Literature based discovery*. It contains additional results and other details which were left out of the main paper to enhance its clarity.

2 Formal Definitions of Evaluation Metrics

1. **Mean Average Precision (MAP):** Given a ranked list of predicted terms (C) relevant to a particular query (A) term, we can calculate the precision after each true positive. The average of these values gives the average precision for that query. This done over all queries gives a single value measure which weights all queries (difficult or easy) equally.

   $MAP = \frac{\Sigma_i AP(i)}{|V|}$,
   where $|V|$ = number of queries, AP(i) = $\Sigma_n(R_n - R_{n-1})P_n$ and P_n and R_n are the Precision and Recall at the nth threshold for the ith query.

2. **Mean Reciprocal Rank (MRR):**

   $\Sigma_i \frac{1}{rank(i)}$,
   where $rank(i)$ = absolute rank for the ith query.

3. **Averaged R(elevant)-Precision:** Similar to MAP but instead of calculating the precision after each positive term (gold C) in the list of results for a given query, precision is only calculated with the top *R* results. *R* is determined by how many true positives exist for the query. The main difference from MAP is that this metric does not consider the remainder of the ranked list outside of the length of the top *R*. This also gives a single value measure which weights all queries equally. This metric is similar to precision at *k* except that instead of having a fixed *k*, it changes based on the amount of positives each node has so that a query with less than *k* positives is not unfairly penalised and a query with a lot more positives than *k* is not easier for the approach to perform well at.

   $AveragedR - precision = \frac{\Sigma_i Pr@R(i)}{|V|}$,

   where $|V|$ = number of nodes, $Pr@R(i)$ = precision at R for the ith node with R positives.

3 Other neural network hyperparameters

LINE: learning rate = 0.025, number of negative samples = 5 and total number of samples = 1 billion. According to (Tang et al., 2015), LINE performs best when it is run twice to obtain first- and second-order proximity embeddings which are concatenated and L2 normalized. I follow their recommendations. For each order I created half the number of dimensions as needed so that when they were concatenated, the final result had the appropriate number.

4 Node combination methods

A neural network approach to LBD with node embeddings requires the model input to be a single vector so the embeddings of the nodes involved in a link need to be combined. This can be done in several ways. Concatenating the embeddings is simple and preserves all information but doubles the size of the input. (Grover and Leskovec, 2016) used four methods which preserve the input size and we experimented with all five methods, detailed in Table 1.

Operator	Definition
Average	$\frac{f_i(u)+f_i(v)}{2}$
Concatenate	$f(u)\,.\,f(v)$
Hadamard	$f_i(u) * f_i(v)$
Weighted-L1	$\|f_i(u) - f_i(v)\|$
Weighted-L2	$\|f_i(u) - f_i(v)\|^2$

Table 1: Node Combination methods. Binary operators are element-wise.

4.1 The LION Test Cases and Evaluation

These cases are described in detail in (Pyysalo et al., 2018). A condensed version is presented here for completeness.

To identify discoveries, the cancer researchers involved in the project first surveyed articles published between 2006 and 2016 in journals that publish works pertaining to biomolecular cancer, such as Science, Nature, The Lancet, British Journal of Cancer, and Cell. In the initial pass, they sought to identify specific cancer-related discoveries that can be characterized as a causal chain of three concepts, i.e. that fit the constraints of the traditional ABC paradigm of LBD. This initial literature survey yielded 50 candidate discoveries. The second stage filtered the candidates to identify discoveries that could have potentially been found by LBD: the two connections A-B and B-C should be found in the literature at some point in time before the connection between A and C is published. They identified cases where in some year in the past, A-B and B-C each co-occurred in at least 100 publications but where no or very few publications had A-C co-occur. To avoid possible bias towards a particular NLP methods or LBD tools the filtering was performed manually using PubMed searches. In this manner the 50 candidates were culled to 16 which were then assessed by all project participants. This yielded a final set of 5 triples that represented specific recent discoveries on the molecular biology of cancer that could have potentially been suggested by an LBD system prior to their publication. The ontology and database identifier in the relevant resources were manually identified for each of the concepts in the dataset. In addition to these 5 cancer cases, in an effort to continue the trend of prior work, 5 cases from Swanson were also evaluated by the system. Details of these can be found in Table 2 which is adapted from (Pyysalo et al., 2018).

5 Results

The results of the neural approaches are means of the means which were calculated over 5 runs. The standard deviations reported are of the mean ranks. The results of the baselines are means of the method across all relevant cases and the standard deviations are those over those ranks. The best rank is in **boldface** type. We sought to determine what methods gave the lowest mean ranks and lowest variance (measured by standard deviation). Where necessary, we use results from (Pyysalo et al., 2018).

Wherever there are models that do not use aggregators or accumulators, the results are simply placed in the first column - this is merely for convenience, the column headers *would not* apply to such models. The best for a particular approach is underlined while the best of all approaches is in **bold**.

There were some experiments which produced ties with the gold which were of an amount to make them useless for real-world use. We defined that number as 10; methods which produced more than 10 ties with the gold are reported with a '*' instead of their performance.

A	B	C	Reference
NF-κB	Bcl-2	Adenoma	(Van Der Heijden et al., 2016)
NOTCH1	senescence	C/EBPβ	(Hoare et al., 2016)
IL-17	p38α	MKP-1	(Gaffen and McGeachy, 2015)
Nrf2	ROS	pancreatic cancer	(DeNicola et al., 2011)
CXCL12	senescence	thyroid cancer	(Kim et al., 2017)
Migraine	-	Magnesium	(Swanson, 1988)
Somatomedin C	-	Arginine	(Swanson, 1990)
Alzheimers Disease	-	Estrogen	(Smalheiser and Swanson, 1996b)
Alzheimers Disease	-	Indomethacin	(Smalheiser and Swanson, 1996a)
Schizophrenia	-	Calcium Independent Phospholipase A_2	(Smalheiser and Swanson, 1998)

Table 2: The Cancer Discovery and Swanson cases used to evaluate the LION System.

5.1 Cancer Discoveries and Swanson Cases

Results for Closed Discovery performed on the 5 Cancer discovery cases on which LION was originally evaluated are in Tables 3 and 4.

Results for Open Discovery performed on the 5 Cancer Discovery cases on which LION was evaluated as reported in the paper. Means are in Table 5 and medians are in Table 6.

Results for Open Discovery performed on the 5 Swanson cases on which LION was evaluated. Means are in Table 7 and medians are in Table 8.

Results for Open Discovery performed on the 5 Cancer and 5 Swanson cases on which LION was evaluated. Means are in Table 9 and medians are in Table 10.

Approach	Min	Avg	Max
**NPMI**	278.2	272.6	282.0
**SCP**	252.2	285	298.6
χ^2	268.2	258.0	269.8
***t*-test**	262.0	246.8	260.8
**LLR**	266.0	246.4	264.0
**Jaccard**	214.8	258.8	281.6
**Count**	233.2	249.6	245.2
**Doc-count**	236.8	224.4	222.2
**CD-1-A**	112.9	86.3	97.2
**CD-1-C**	151.2	94.5	89.7
**CD-1-H**	357.2	251.3	287.0
**CD-1-W1**	228.7	195.8	189.0
**CD-1-W2**	614.3	482.9	565.2
**CD-2-A**	86.9	-	-
**CD-2-C**	**48.7**	-	-
**CD-2-H**	143.1	-	-
**CD-2-W1**	402.6	-	-
**CD-2-W2**	63.8	-	-

Table 3: Mean Ranks for Closed Discovery on the Cancer Discovery Cases

5.2 Published Interactions: BioGRID

The results of the BioGRID experiments are in the Tables 11 to 14. Each table is dedicated to a single metric: Mean Rank (MR), Mean Reciprocal Rank (MRR), Mean Average Precision (MAP) and Mean Relevance-precision (R-precision).

Due to rounding, some scores seem equal in the tables but are not. Where this occurs and involves a best performer, the unrounded number was used to break the ties.

**Approach**	**Min**	**Avg**	**Max**
**NPMI**	86.0	119.0	170.0
**SCP**	70.0	196.0	299.0
χ^2	74.0	196.0	270.0
***t*-test**	56.0	136.0	261.0
**LLR**	65.0	163.0	264.0
**Jaccard**	81.0	213.0	282.0
**Count**	245.0	181.0	245.0
**Doc-count**	231.0	169.0	222.0
**CD-1-A**	96.0	93.8	89.4
**CD-1-C**	158.6	**36.4**	38.8
**CD-1-H**	282.8	176.0	238.8
**CD-1-W1**	109.4	158.4	114.8
**CD-1-W2**	300.2	240.0	256.0
**CD-2-A**	52.4	-	-
**CD-2-C**	42.0	-	-
**CD-2-H**	62.2	-	-
**CD-2-W1**	180.6	-	-
**CD-2-W2**	48.8	-	-

Table 4: Median Ranks for Closed Discovery on the Cancer Discovery Cases

**Approach**	**Min**		**Avg**		**Max**	
	**Sum**	**Max**	**Sum**	**Max**	**Sum**	**Max**
**NPMI**	73,670.4	14,658.8	310.2	11,354.6	60.2	3479.2
**SCP**	244.8	2,358.4	553.8	1,408.4	556.0	1,305.4
χ^2	37,387.4	2,971.6	603.4	1,521.2	601.4	1,469.6
***t*-test**	118,606.8	465.6	73,657.2	559.2	126.0	825.0
**LLR**	73,715.0	649.4	253.0	1,011.8	280.4	1,870.8
**Jaccard**	89.2	1,741.8	121.2	952.6	136.2	1,186.0
**Count**	367.4	2,063.6	412.6	1,483.6	421.0	875.8
**Doc-count**	394.4	2,141.8	472.6	1,249.2	490.6	2,071.2
**OD-1-A**	218.3	*	239.1	2,098.0	264.2	*
**OD-1-C**	93.4	*	123.2	37,248.0	156.9	*
**OD-1-H**	257.9	4,762.6	270.6	7,820.9	280.6	*
**OD-1-W1**	212.2	14,932.1	225.1	23,456.7	236.7	*
**OD-1-W2**	247.8	8,777.7	281.48	20,546.9	311.9	*
**OD-2-A**	127.9	-	-	-	-	-
**OD-2-C**	95,207.6	-	-	-	-	-
**OD-2-H**	**31.1**	-	-	-	-	-
**OD-2-W1**	57,226.2	-	-	-	-	-
**OD-2-W2**	586.6	-	-	-	-	-

Table 5: Mean Ranks for Open Discovery on the Cancer Discovery Cases

6 Additional Analyses

The existing approaches performed much better on mean rank for open discovery than they did on closed discovery, so there was more room for improvement there. This lower baseline explains to some degree why the performance improvements were more pronounced for closed discovery (Table 3).

The difference between mean and median as average shows across the various cancer and Swanson discovery cases: with the exception of open discovery on only the Cancer Discovery cases (Tables 5 and

Approach	Min		Avg		Max	
	**Sum**	**Max**	**Sum**	**Max**	**Sum**	**Max**
**NPMI**	98,698.0	15,476.0	121.0	5,897.0	36.0	2,268.0
**SCP**	276.0	926.0	400.0	1,176.0	399.0	727.0
χ^2	547.0	3,582.0	402.0	1,159.0	402.0	1,159.0
***t*-test**	118,751.0	63.0	98,406.0	325.0	125.0	176.0
**LLR**	98,677.0	187.0	344.0	646.0	319.0	645.0
**Jaccard**	29.0	1,089.0	78.0	962.0	93.0	1,122.0
**Count**	15.0	1,005.0	55.0	52.0	62.0	54.0
**Doc-count**	23.0	738.0	72.0	68.0	74.0	68.0
**OD-1-A**	26.8	*	38.6	1,212.6	48.6	*
**OD-1-C**	31.4	*	32.0	30,573.2	34.4	*
**OD-1-H**	46.2	1,750.3	46.6	8,120.4	49.4	*
**OD-1-W1**	28.6	8,905.0	33.4	21,335.2	39.2	*
**OD-1-W2**	43.8	8,370.2	49.0	18,442.8	55.2	*
**OD-2-A**	16.3	-	-	-	-	-
**OD-2-C**	98,148.2	-	-	-	-	-
**OD-2-H**	**12.2**	-	-	-	-	-
**OD-2-W1**	37,268.6	-	-	-	-	-
**OD-2-W2**	147.0	-	-	-	-	-

Table 6: Median Ranks for Open Discovery on the Cancer Discovery Cases

Approach	Min		Avg		Max	
	**Sum**	**Max**	**Sum**	**Max**	**Sum**	**Max**
**NPMI**	39,481.0	12,805.6	27,041.8	13,290.0	4480.4	10,568.6
**SCP**	4,498.8	7,666.0	5,154.8	3,024.0	5,174.8	2,700.4
χ^2	37,873.6	10,402.8	5,182.6	4,702.8	5,319.6	3,803.2
***t*-test**	46,240.0	7,076.2	37,344.2	7,989.2	3,956.4	6,756.2
**LLR**	37,440.8	6,761.6	3,286.6	2,663.0	4,367.4	2,691.0
**Jaccard**	3,179.6	3,629.2	4,342.8	4,105.4	4,455.2	3,878.8
**Count**	3,484.2	2,882.2	4,242.0	2,216.0	4,265.2	5,364.4
**Doc-count**	3,470.8	2,871.0	4,229.6	2,199.8	4,255.6	5,365.2
**OD-1-A**	3,643.0	6,468.8	3,726.7	7,405.2	3,805.3	*
**OD-1-C**	3,721.4	11,229.8	3,757.4	16,325.9	3,788.6	*
**OD-1-H**	3,558.3	*	3,618.0	5,427.8	3,666.5	*
**OD-1-W1**	3,752.8	*	3,928.6	12,814.2	4,058.1	*
**OD-1-W2**	3,746.7	10,100.4	4,091.0	12,183.3	4,345.4	*
**OD-2-A**	6,859.0	-	-	-	-	-
**OD-2-C**	38,639.0	-	-	-	-	-
**OD-2-H**	**1,013.4**	-	-	-	-	-
**OD-2-W1**	29,960.9	-	-	-	-	-
**OD-2-W2**	14,697.4	-	-	-	-	-

Table 7: Mean Ranks for Open Discovery on the Swanson Discovery Cases

6), the best performer for mean and median were different.

A conclusion to be drawn from all the results tables is that although the best neural network-based approaches performed the best, simply using neural networks is not sufficient to produce the best results as there are several instances where the best existing approaches outperformed some neural approaches.

Approach	Min		Avg		Max	
	**Sum**	**Max**	**Sum**	**Max**	**Sum**	**Max**
**NPMI**	41,837.0	8,869.0	16,714.0	9715.0	74.0	5,545.0
**SCP**	124.0	427.0	154.0	250.0	154.0	250.0
χ^2	37,827.0	7,820.0	156.0	263.0	155.0	263.0
***t*-test**	40,103.0	1,808.0	37,368.0	116.0	5.0	105.0
**LLR**	37,820.0	3,404.0	9.0	45.0	10.0	43.0
**Jaccard**	6.0	1,075.0	6.0	237.0	9.0	240.0
**Count**	8.0	43.0	20.0	29.0	21.0	261.0
**Doc-count**	7.0	21.0	20.0	31.0	21.0	237.0
**OD-1-A**	18.4	4,852.3	16.2	6,776.2	18.6	*
**OD-1-C**	**4.0**	1,917.8	9.6	6,933.0	16.4	*
**OD-1-H**	19.2	*	14.2	6,173.2	13.4	*
**OD-1-W1**	17.6	*	19.8	1,907.2	20.4	*
**OD-1-W2**	25.0	2,570.6	22.6	2,546.6	21.8	*
**OD-2-A**	605.4	-	-	-	-	-
**OD-2-C**	37,783.8	-	-	-	-	-
**OD-2-H**	17.6	-	-	-	-	-
**OD-2-W1**	44,254.0	-	-	-	-	-
**OD-2-W2**	49.6	-	-	-	-	-

Table 8: Median Ranks for Open Discovery on the Swanson Discovery Cases

Approach	Min		Avg		Max	
	**Sum**	**Max**	**Sum**	**Max**	**Sum**	**Max**
**NPMI**	56,575.7	13,732.2	13,676	12,322.3	2,270.3	7,023.9
**SCP**	2,371.8	5,012.2	2,854.3	2,216.2	2,865.4	2,002.9
χ^2	37,630.5	6,687.2	2,893.0	3,112.0	2,960.5	2,636.4
***t*-test**	82,423.4	3,770.9	55,500.7	4,274.2	2,041.2	3,790.6
**LLR**	55,577.9	3,705.5	1,769.8	1,837.4	2,323.9	2,280.9
**Jaccard**	1,634.4	2685.5	2,232.0	2,529.0	2,295.7	2,532.4
**Count**	1,925.8	2,472.9	2,327.3	1,849.8	2,343.1	3,120.1
**Doc-count**	1,932.0	2,506.4	2,351.1	1,724.5	2,373.1	3,718.2
**OD-1-A**	1,930.7	*	1,982.9	4,751.6	2,034.8	*
**OD-1-C**	1,907.42	*	1,940.3	26,786.9	1,972.8	*
**OD-1-H**	1,908.08	*	1,944.28	6,624.36	1,973.5	*
**OD-1-W1**	1,982.5	*	2,076.86	18,135.42	2,147.4	*
**OD-1-W2**	1,997.3	9,439.0	2,186.2	16,365.1	2,328.7	*
**OD-2-A**	3,493.5	-	-	-	-	-
**OD-2-C**	66,923.3	-	-	-	-	-
**OD-2-H**	**522.2**	-	-	-	-	-
**OD-2-W1**	43,593.5	-	-	-	-	-
**OD-2-W2**	7,642.0	-	-	-	-	-

Table 9: Mean Ranks for Open Discovery on the all Cases

References

Gina M DeNicola, Florian A Karreth, Timothy J Humpton, Aarthi Gopinathan, Cong Wei, Kristopher Frese, Dipti Mangal, H Yu Kenneth, Charles J Yeo, Eric S Calhoun, et al. 2011. Oncogene-induced Nrf2 transcription promotes ROS detoxification and tumorigenesis. *Nature*, 475(7354):106.

Sarah L Gaffen and Mandy J McGeachy. 2015. Integrating p38α MAPK immune signals in nonimmune cells. *Sci. Signal.*, 8(366):fs5–fs5.

Approach	Min		Avg		Max	
	**Sum**	**Max**	**Sum**	**Max**	**Sum**	**Max**
**NPMI**	50,347.0	10,624.0	698.0	9,472.0	55.0	3,630.0
**SCP**	200.0	758.5	370.5	9,472.0	371.0	630.0
χ^2	35,808.5	3,712.5	379.5	958.0	380.5	873.5
***t*-test**	78,806.0	344.5	48,107	220.5	43.5	169.5
**LLR**	48,337.0	569.0	44.5	420.0	46.5	540.5
**Jaccard**	21.0	1,082.0	46.5	610.5	57.5	849.0
**Count**	**11.5**	285.0	46.5	610.5	57.5	849.0
**Doc-count**	12.5	237.0	44.5	60.0	47.5	152.5
**OD-1-A**	25.2	*	29.2	3,850.4	32.7	*
**OD-1-C**	18.2	*	21.8	23,334.8	26.6	*
**OD-1-H**	22.4	*	23.4	6,901.6	25.3	*
**OD-1-W1**	20.3	*	21.2	19,214.2	25.1	*
**OD-1-W2**	28.2	5,470.4	27.3	13,559.3	30.4	*
**OD-2-A**	399.6	-	-	-	-	-
**OD-2-C**	54,867.2	-	-	-	-	-
**OD-2-H**	14.9	-	-	-	-	-
**OD-2-W1**	40,761.3	-	-	-	-	-
**OD-2-W2**	98.3	-	-	-	-	-

Table 10: Median Ranks for Open Discovery on all Cases

Approach	Min		Avg		Max	
	**Sum**	**Max**	**Sum**	**Max**	**Sum**	**Max**
**NPMI**	1,211.9	1,675.4	1,173.9	1,692.8	1,156.5	1,657.4
**SCP**	1,342.8	1,616.5	1,291.7	1,585.4	1,293.1	1,558.8
χ^2	1,376.1	1,623.0	1,305.0	1,591.1	1,304.2	1,564.3
***t*-test**	1,172.1	1,423.1	1,163.8	1,320.1	1,149.9	1,301.9
**LLR**	1,205.8	1,496.1	1,137.8	1,358.1	1,132.9	1,326.4
**Jaccard**	1,197.3	1,547.1	1,178.5	1,477.0	1,169.9	1,431.5
**Count**	1,175.4	1,659.0	1,146.0	1,335.6	1,146.0	1,341.6
**OD-1-A**	1,911.5	1,912.0	1,909.5	1,909.5	1,908.5	1,911.7
**OD-1-C**	1,910.5	1,909.6	1,909.5	1,909.5	1,913.4	1,915.8
**OD-1-H**	1,914.3	1,912.8	1,909.5	1,909.5	1,907.5	1,910.6
**OD-1-W1**	1,910.6	1,910.3	1,909.5	1,909.5	1,908.3	1,911.6
**OD-1-W2**	1,910.3	1,910.5	1,909.5	1,909.5	1,908.3	1,914.0
**OD-2-A**	1,154.1	-	-	-	-	-
**OD-2-C**	**1,113.1**	-	-	-	-	-
**OD-2-H**	1,315.8	-	-	-	-	-
**OD-2-W1**	1,670.4	-	-	-	-	-
**OD-2-W2**	1,869.5	-	-	-	-	-

Table 11: Mean Mean Ranks (MR) for time-sliced BioGRID

Aditya Grover and Jure Leskovec. 2016. node2vec: Scalable feature learning for networks. In *Proceedings of the 22nd ACM SIGKDD International Conference on Knowledge Discovery and Data Mining*.

Matthew Hoare, Yoko Ito, Tae-Won Kang, Michael P Weekes, Nicholas J Matheson, Daniel A Patten, Shishir Shetty, Aled J Parry, Suraj Menon, Rafik Salama, et al. 2016. NOTCH1 mediates a switch between two distinct secretomes during senescence. *Nature Cell Biology*, 18(9):979.

Young Hwa Kim, Yong Won Choi, Jeonghun Lee, Euy Young Soh, Jang-Hee Kim, and Tae Jun Park. 2017. Senescent tumor cells lead the collective invasion in thyroid cancer. *Nature Communications*, 8:15208.

**Approach**	**Min**		**Avg**		**Max**	
	**Sum**	**Max**	**Sum**	**Max**	**Sum**	**Max**
**NPMI**	2.75	1.40	2.62	1.47	2.35	0.96
**SCP**	2.29	1.48	1.80	1.36	1.51	0.98
χ^2	2.25	1.47	1.79	1.36	1.50	0.98
***t*-test**	2.51	1.87	2.52	1.33	2.37	1.10
**LLR**	2.83	1.79	2.19	1.27	1.90	1.10
**Jaccard**	<u>2.86</u>	1.44	2.57	1.43	2.12	1.04
**Count**	2.00	1.04	1.91	1.07	1.70	0.94
**OD-1-A**	1.22	1.08	1.27	1.27	1.26	1.25
**OD-1-C**	1.25	1.14	1.27	1.27	1.24	1.20
**OD-1-H**	1.24	1.12	1.27	1.27	1.25	1.24
**OD-1-W1**	1.21	1.11	1.27	1.27	1.26	1.25
**OD-1-W2**	1.21	1.11	1.27	1.27	<u>1.26</u>	1.25
**OD-2-A**	5.17	-	-	-	-	-
**OD-2-C**	**5.46**	-	-	-	-	-
**OD-2-H**	4.11	-	-	-	-	-
**OD-2-W1**	2.58	-	-	-	-	-
**OD-2-W2**	2.46	-	-	-	-	-

Table 12: Mean Mean Average Precision (MAP) for time-sliced BioGRID

**Approach**	**Min**		**Avg**		**Max**	
	**Sum**	**Max**	**Sum**	**Max**	**Sum**	**Max**
**NPMI**	2.08	1.14	1.96	1.2	1.81	0.82
**SCP**	1.7	1.21	1.35	1.08	1.23	0.83
χ^2	1.68	1.21	1.34	1.08	1.23	0.83
***t*-test**	1.88	1.61	1.9	0.96	1.82	0.82
**LLR**	2.17	1.63	1.56	0.92	1.34	0.81
**Jaccard**	<u>2.19</u>	1.19	1.96	1.15	1.66	0.86
**Count**	1.77	1.04	1.49	0.9	1.3	0.78
**OD-1-A**	0.92	0.82	0.92	0.92	0.93	0.93
**OD-1-C**	0.92	0.85	0.92	0.92	<u>0.94</u>	0.93
**OD-1-H**	0.93	0.86	0.92	0.92	0.92	0.92
**OD-1-W1**	0.90	0.85	0.92	0.92	0.92	0.91
**OD-1-W2**	0.91	0.84	0.92	0.92	0.92	0.91
**OD-2-A**	3.36	-	-	-	-	-
**OD-2-C**	**3.42**	-	-	-	-	-
**OD-2-H**	2.78	-	-	-	-	-
**OD-2-W1**	1.75	-	-	-	-	-
**OD-2-W2**	1.76	-	-	-	-	-

Table 13: Mean Mean Reciprocal Rank (MRR) for time-sliced BioGRID

Sampo Pyysalo, Simon Baker, Imran Ali, Stefan Haselwimmer, Tejas Shah, Andrew Young, Yufan Guo, Johan Hgberg, Ulla Stenius, Masashi Narita, and Anna Korhonen. 2018. LION LBD: a literature-based discovery system for cancer biology. *Bioinformatics*, page bty845.

Neil R Smalheiser and Don R Swanson. 1996a. Indomethacin and Alzheimer's disease. *Neurology*, 46(2):583–583.

Neil R Smalheiser and Don R Swanson. 1996b. Linking estrogen to Alzheimer's disease an informatics approach. *Neurology*, 47(3):809–810.

Approach	Min		Avg		Max	
	**Sum**	**Max**	**Sum**	**Max**	**Sum**	**Max**
**NPMI**	2.35	1.0	2.06	1.03	1.89	0.56
**SCP**	1.95	1.08	1.24	0.86	1.01	0.58
χ^2	1.89	1.08	1.23	0.86	1.0	0.58
***t*-test**	1.9	1.4	1.94	0.86	1.8	0.74
**LLR**	2.47	1.52	1.75	0.68	1.38	0.58
**Jaccard**	2.47	1.05	2.18	0.89	1.64	0.54
**Count**	1.9	0.79	1.44	0.73	1.35	0.62
**OD-1-A**	0.96	0.83	0.98	0.98	1.00	0.99
**OD-1-C**	0.97	0.88	0.98	0.98	1.01	1.01
**OD-1-H**	1.00	0.88	0.98	0.98	0.96	0.96
**OD-1-W1**	0.95	0.89	0.98	0.98	0.97	0.97
**OD-1-W2**	0.97	0.86	0.98	0.98	0.98	0.97
**OD-2-A**	4.45	-	-	-	-	-
**OD-2-C**	**4.73**	-	-	-	-	-
**OD-2-H**	3.78	-	-	-	-	-
**OD-2-W1**	2.15	-	-	-	-	-
**OD-2-W2**	1.87	-	-	-	-	-

Table 14: Mean Relevance-precision (R-precision) for time-sliced BioGRID

Neil R Smalheiser and Don R Swanson. 1998. Calcium-independent Phospholipase A2 and Schizophrenia. *Archives of General Psychiatry*, 55(8):752–753.

Don R Swanson. 1988. Migraine and Magnesium: eleven neglected connections. *Perspectives in Biology and Medicine*, 31(4):526–557.

Don R Swanson. 1990. Somatomedin C and Arginine: implicit connections between mutually isolated literatures. *Perspectives in Biology and Medicine*, 33(2):157–186.

Jian Tang, Meng Qu, Mingzhe Wang, Ming Zhang, Jun Yan, and Qiaozhu Mei. 2015. LINE: Large-scale Information Network Embedding. In *Proceedings of WWW 2015*. ACM.

Maartje Van Der Heijden, Cheryl D Zimberlin, Anna M Nicholson, Selcuk Colak, Richard Kemp, Sybren L Meijer, Jan Paul Medema, Florian R Greten, Marnix Jansen, Douglas J Winton, et al. 2016. Bcl-2 is a critical mediator of intestinal transformation. *Nature Communications*, 7:10916.
